# Supplementary material for: Analysis of Let-7 Family miRNA in Plasma as Potential Predictive Biomarkers of Diagnosis for Papillary Thyroid Cancer
Source: Diagnostics (Basel). 2020 Feb 28;10(3):130. doi: 10.3390/diagnostics10030130 (PMC7151036; doi:10.3390/diagnostics10030130)
Supplement: Supplementary file 1 [file diagnostics-10-00130-s001.pdf]

Sample no	Gender	Age	let-7i	let-7a	let-7c	let-7f	let-7d
1	F	29	10,6	12,2	14,9	6,3	2,7
2	M	56	46,5	27,5	25	11,1	6,2
3	M	53	2,4	7,3	4,1	6,1	1,5
4	M	54	40,5	24,9	21,4	11,1	5,1
5	M	44	6,4	5,7	2,2	2,5	1,1
6	F	46	20	17,9	12,8	8,1	2,5
7	F	61	22,8	25,4	13,5	3,5	3,3
8	F	55	83,7	41,5	22,1	12	14,4
9	M	53	72,4	97	42	34,8	13,7
10	M	42	31,6	27,7	22,8	8,9	4,5
11	F	42	19,5	14	17,1	6,5	2,4
12	M	44	12,4	14,8	12,1	3,2	2,2
13	F	55	86	55,5	45,7	15,7	17,9
14	F	67	39,1	68	42	13,7	8,4
15	F	44	16,8	13	17	4,6	3,5
16	M	44	34	24,2	27,6	9,8	6,6
17	F	46	91,8	2	9,1	1,1	5
18	F	47	250	186	173	59,6	77,5
19	F	66	181	255	146	78,7	84,2
20	F	59	147	185	117	73,7	61
21	M	40	21,2	44,1	8,5	15,2	2,5

Table S1. Characteristics of controls.

Sample no	Gender	Age	T	N	let-7i	let-7a	let-7c	let-7f	let-7d
22	M	33	T1	N1	76	36,9	45	9,7	9,1
23	F	55	T1	N0	346	453	195	145	151
24	M	50	T3	NX	43,6	21,7	73,4	7,7	5
25	F	32	T3	N0	122	38,8	202	15,5	15,2
26	F	70	T3	N0	178	127	128	44,8	38,1
27	F	59	T1	N1	177	127	275	35,7	34,7
28	F	42	T2	N0	410	231	176	91	113
29	F	74	T1	N0	77	54,3	57,3	22,7	15,6
30	F	76	T1	N1	188	133	232	47,5	34
31	F	56	T1	N1	411	488	195	130	153
32	F	44	T1	N1	430	636	123	193	217
33	F	67	T1	N0	222	109	174	41,3	41,6
34	F	63	T1	N0	336	388	395	147	133
35	F	39	T3	N0	343	453	153	139	145
36	F	71	T3	N0	336	77,5	279	29,3	34,8
37	F	66	T3	N1	3220	5450	475	1294	1096
38	F	51	T1	N1	49,1	29,6	45,4	10,6	6,9
39	F	37	T2	N0	1180	301	258	82,7	98
40	F	50	T1	N0	103	77,3	75,7	16,5	28
41	F	48	T1	N0	184	104	84,6	41,1	35,2
42	F	56	T1	N1	42,6	35,6	21,7	6,8	7,9
43	F	57	T1	N0	22,3	11,1	32,6	3,8	2,6
44	F	28	T1	N0	188	127	103	42,4	35,2
45	F	30	T1	N0	56,2	59,2	51,3	19,2	12,7
46	F	47	T1	N0	3,4	8,4	5	10,9	1,8
47	F	29	T1	N0	12	12,7	8,8	3,7	2,5
48	F	49	T1	N1	10,7	3,2	3,3	2	1
49	F	25	T1	N0	33,5	64,6	22,5	18,1	13
50	F	46	T1	N0	7,9	10,3	5,8	5,9	2,5
51	F	58	T1	N0	43,5	93	16	35,2	23,2
52	F	59	T1	NX	62,7	22,3	50,5	18,6	14,2
53	F	67	T1	NX	13,4	28,5	21,5	14,2	9,8
54	M	29	T2	NX	73	61,6	20,2	24,5	21,1
55	F	38	T1	N0	457	166	394	57,6	45,5
56	M	55	T3	N0	38,2	46,4	13,5	16,2	10,7
57	F	29	T1	NX	23	185	51,6	90	61,1
58	M	74	T4	NX	11,1	21,6	7,8	7,7	3,6
59	F	24	T1	N0	36,3	35,6	16,7	9,9	5,8
60	F	20	T3	N1	4,4	3,3	2,4	3	1,6
61	F	53	T1	N0	11,5	16,2	6,8	9,4	6,8
62	F	41	T3	N1	11,1	14,1	5,2	9	3,5
63	F	48	T2	N0	67	65,7	50,5	19,5	13,3
64	F	46	T2	N0	26,6	17,1	14,7	6,9	4,1
65	F	73	T1	NX	5,4	7,1	12,6	6,5	1,7
66	F	49	T3	N1	22,8	53,1	17,5	8	5,8
67	F	40	T1	N0	26	149	17,5	9,2	6,4
68	F	30	T1	NX	246	82,3	54,3	78	47,4
69	F	38	T3	N1	129	205	65,4	29,8	24,7
70	F	42	T1	N0	256	29,1	144	61,7	34,1

Table S2. Characteristics of PTC cases.

Let-7	Sensitivity	Specificity
Let-7a+ Let-7c	53	14
Let-7a+ Let-7f	59	19
Let-7a+ Let-7d	71	33
Let-7c+ Let-7f	65	28
Let-7c+ Let-7d	55	14
Let-7f+ Let-7d	59	23

Table S3. Combinations of the let-7 miRNAs.
